# Case Report: Amphiphysin Antibody-Associated Stiff-Limb Syndrome and Myelopathy: An Unusual Presentation of Breast Cancer in an Elderly Woman

**DOI:** 10.3389/fneur.2021.735895

**Published:** 2021-10-28

**Authors:** Bhanu Gogia, Elena Shanina, Xiang Fang, Jing He, Xiangping Li

**Affiliations:** ^1^Department of Neurology, Beth Israel Deaconess Medical Center/Harvard Medical School, Boston, MA, United States; ^2^Department of Neurology, University of Texas Medical Branch, Galveston, TX, United States; ^3^Department of Pathology, University of Texas Medical Branch, Galveston, TX, United States

**Keywords:** stiff person syndrome, stiff limb syndrome, amphiphysin antibody, neurologic paraneoplastic syndromes, paraneoplastic myelopathy

## Abstract

**Background:** Paraneoplastic stiff-limb syndrome (SLS) is a rare manifestation of underlying malignancy and could have distinctive features different from the classic stiff-person syndrome (SPS).

**Case Description:** We present a case of anti-amphiphysin antibody (Ab)-associated paraneoplastic SLS, in an 83-year-old woman with invasive ductal carcinoma of the breast. She presented with stiffness, painful spasms of the distal legs, and asymmetrical fixed posturing of the foot. There are coexisting long-tract disturbance and lower-extremity weakness. Treatment with diazepam provided symptomatic relief while plasma exchange (PLEX) did not lead to significant clinical improvement. The patient was bedridden within 3 months and passed away within 6 months from symptom onset.

**Conclusion:** This case highlights the importance of recognition of uncommon presentation of SPS and its oncological significance. This entity requires a high degree of suspicion for initiation of the proper workup. The rapid identification and treatment of the underlying tumor might offer the best chance for recovery.

## Introduction

More than half a century has passed since classic stiff-person syndrome (SPS) was first described in 1956 by Moersch and Woltman in a case series of 14 patients at the Mayo Clinic (1). Classic SPS is a rare neuroimmunological disorder that is characterized by symmetrical muscle stiffness and painful spasms affecting the axial and limb muscles, without extrapyramidal or pyramidal tract signs. SPS is currently considered as a spectrum disorder including classic SPS, paraneoplastic SPS, and SPS variants. SPS variants include focal forms like stiff-limb syndrome (SLS), jerking SPS, progressive encephalomyelitis with rigidity and myoclonus (PERM), and SPS plus (ataxia, epilepsy, etc.) (2). SPS has been linked most commonly to anti-GAD 65 (glutamic acid decarboxylase, 70–80%) and less commonly to anti-GlyR (anti-glycine receptor, 10%), anti-amphiphysin (5%), anti-DPPX (anti-dipeptidyl-peptidase-like protein), anti-gephyrin, and anti-GABAaR antibodies (Abs) (3).

Paraneoplastic SPS occurs in 5–10% of all patients with SPS and is frequently associated with underlying malignancies of breast, lung, colon, thymus, and Hodgkin's lymphoma (3, 4). Anti-amphiphysin Ab is the most common marker of this variant most commonly associated with breast cancer. Paraneoplastic neurologic syndromes occur as a result of immune cross-reactivity between the tumor and host cells. In 80% of the cases, paraneoplastic neurologic syndromes can precede a tumor diagnosis and can help detect an occult malignancy (5). The diagnosis of SPS is challenging given its heterogeneity in symptomatology, clinical course, and presence of autoimmune Abs.

We hereby present a case of rapid progressive paraneoplastic SLS with the coexistence of myelopathic features. These distinctive clinical features are extremely uncommon and therefore will contribute to the pool of literature to understand this rare entity.

## Case Presentation

An 83-year-old white female with a medical history of hypertension, celiac disease, and gout presented with bilateral lower-extremity weakness and painful spasms for 3 months, which were worsening over 2 weeks. She had spasms in the feet, causing dystonic posturing resembling “clubfoot,” and she was unable to straighten them or bend her knees. She also endorsed numbness, primarily on the left foot. She initially used a cane for ambulation when her symptoms started but later used a wheelchair. She denied any bowel or bladder incontinence, but due to restricted mobility, she was using diapers. About 6 weeks before the presentation, the patient noted swelling in the bilateral lower extremity and was prescribed steroids for a presumed gout flare. However, the spasms and pain worsened, and the swelling did not resolve. There was also reduced appetite, which caused weight loss of ~20 lb in the month prior to presentation. She had regular mammograms in the past. She reported that her last mammogram at 70 years old was abnormal but could not provide any specifics. She noticed a painless left-breast mass, which grew progressively in size slowly. She did not pursue any further evaluation given her age. She denied use of tobacco, alcohol, or illicit drugs. Family history indicated that her two sisters were both diagnosed with breast cancer in their 60's.

During the examination, she was alert and oriented. Cranial nerves were intact. She had normal strength and reflexes in the upper extremities (UE). Strength was 3/5 in the left lower extremity and 4+/5 in the right lower extremity proximally but 3/5 in ankle dorsiflexion and plantar flexion. Patellar reflexes were normal, and ankle reflexes were absent bilaterally.

**Figure 1:**
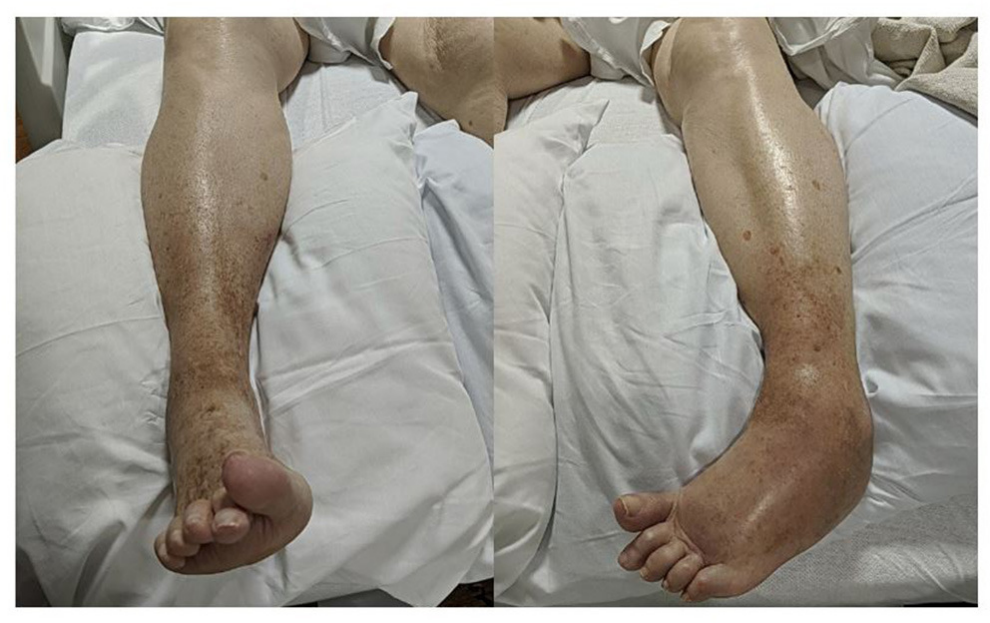
Asymmetrical dystonic posturing on both sides (left more than right) with significant swelling and redness in the dorsum of the left foot.

Pinprick sensation, proprioception, and vibration were diminished in the left lower extremity up to the ankle, but there was no sensory level or saddle anesthesia. She had dystonic posturing of bilateral feet (left more than right), with significant swelling and redness in the dorsum of the left foot (Figure 1). Babinski signs were present bilaterally. She had multiple intermittent spasms in both lower limbs distally, triggered by mild tactile stimuli and causing significant pain. The breast exam showed a 5 × 6-cm firm palpable mass in the upper outer quadrant of the left breast and a left axillary firm, non-tender, and enlarged lymph node.

**Figure 2:**
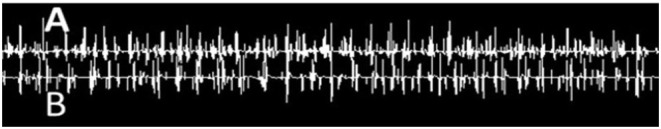
Needle EMG recording of simultaneous co-activation of agonist–antagonist muscle pair using a concentric needle electrode and two-channel recording from **(A)** tibialis anterior and **(B)** gastrocnemius muscles.

Brain and whole-spine magnetic resonance imaging (MRI) were unremarkable. Cerebrospinal fluid (CSF) showed mild lymphocytic pleocytosis, elevated protein level (white blood cell [WBC] 6, red blood cell [RBC] 53, protein 66, and glucose 52), and oligoclonal bands (CSF-restricted). The CSF meningitis panel was negative. Creatinine kinase was transiently elevated (1,253 U/L on admission) with an elevated erythrocyte sedimentation rate (ESR) of 40 mm/h and C-reactive protein (CRP) of 14.6 mg/dl. The patient was started on multiple muscle relaxants including methocarbamol, cyclobenzaprine, and baclofen with minimal response. Electromyography (EMG) showed sensorimotor axonal polyneuropathy in lower extremities, as well as continuous motor unit activity and co-activation of agonists and antagonist muscles (Figure 2), typical for SPS.

Following the EMG, the patient was prescribed diazepam, which relieved the painful spasms. Due to the reported depression from prior steroid use and newly diagnosed diabetes (HbA1C 7.2), methylprednisolone was not initiated. She was treated with five sessions of plasma exchange (PLEX). Serum amphiphysin Ab was identified as positive in the Autoimmune Neurologic Disease Panel and Paraneoplastic Reflexive Panel (ARUP) laboratory, but the titer was not available. The GAD 65 Ab result was negative. Other relevant laboratories revealed normal vitamin B12 level (782 pg/ml), as well as negative thyroid peroxidase Ab (TPO), thyroglobulin Ab, gangliosides, and polymyositis panel (Table 1).

**Table 1:** Summary of laboratory testing.

**Laboratory work**	**Result**	**Reference interval**
Amphiphysin	Positive	Negative
Glutamic acid decarboxylase antibody 65 (GAD 65)	<5.0 IU/ml	0.0–5.0
Purkinje cell/neuronal nuclear IgG	None detected	None detected
Striated muscle antibodies	<1:40	<1:40
NMDA receptor antibody	<1:10	<1:10
CV2.1 antibody	<1:10	<1:10
Acetylcholine binding antibody	0.0 nmol/L	0.0–0.4
Voltage-gated calcium channel (VGCC) antibody	0.0 nmol/L	0.0–24.5
Aquaporin-4 receptor antibody	1.0 U/ml	≤ 2.9
Voltage-gated potassium channel antibody	0.0 nmol/l	0–31
Titin antibody	<0.09 IV	0.00–0.45
Gastric parietal cell antibody	1.8 units	0.0–24.9
Ganglioside panel	Asialo-GM1, GM1, GM2, GD1a, GD1b, and GQ1b antibodies unremarkable	0–50
Thyroid peroxidase antibody (TPO)	<0.3 IU/ml	0.0–9.0
Thyroglobulin antibody	<0.9 IU/ml	0.0–4.0
Vitamin B12	782 pg/ml	240–930
Folic acid	8.9 ng/ml	3.0–20.0
Cancer antigen 27.29	<3.5 U/ml	0.0–40.0
CSF oligoclonal bands	Positive (4 bands)	Negative (0–1)
CSF meningitis/encephalitis panel by PCR	*Escherichia coli, Haemophilus influenzae, Listeria monocytogenes, Neisseria meningitidis, Streptococcus agalactiae, Streptococcus pneumoniae*, Cytomegalovirus, Enterovirus, Herpes simplex virus 1 and 2, Human herpesvirus 6, Human parechovirus, Varicella zoster virus, and Cryptococcus neoformans negative	Negative

**Figure 3:**
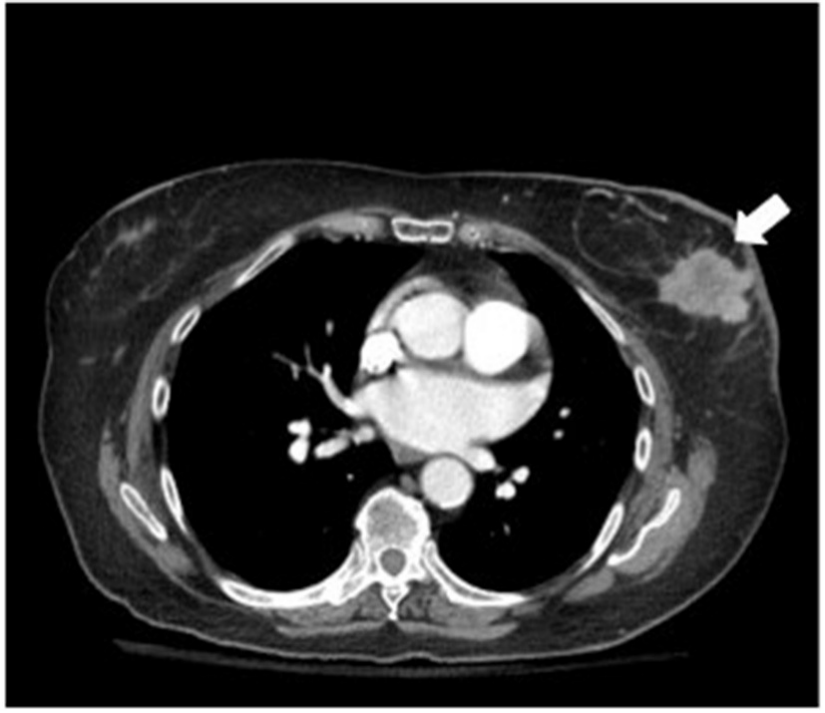
Chest CT showed a 4-cm left-breast mass with central necrosis.

**Figure 4:**
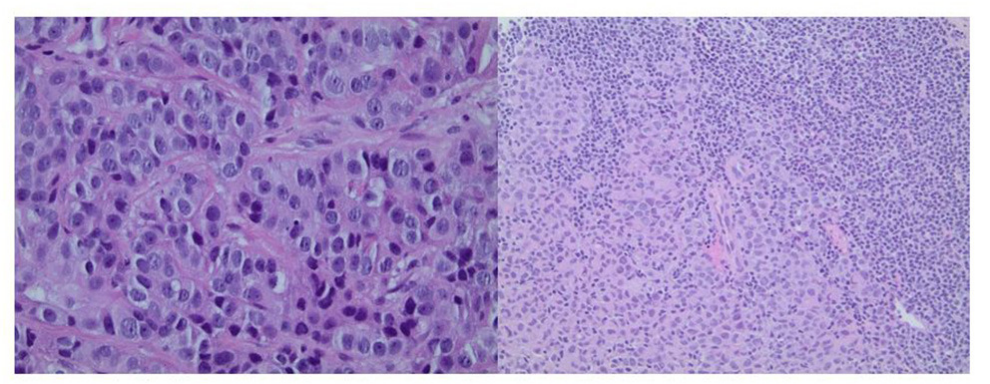
Left panel: Core biopsy section from the left-breast mass shows invasive ductal carcinoma (×400 magnification). Right panel: Core biopsy section from the left infraclavicular lymph node shows metastatic breast carcinoma cells in the lymphoid tissue (×200 magnification).

A computed tomography (CT) scan of the chest, abdomen, and pelvis showed a 4-cm left-breast mass with central necrosis and multiple large left axillary lymph nodes (Figure 3). Left-breast mass and the left infraclavicular lymph node biopsy revealed invasive ductal carcinoma with metastatic breast carcinoma cells in the lymphoid tissue (Figure 4), Stage IIIC. Neoplastic cells were positive for estrogen and progesterone receptor staining but negative for Her2 protein. Oncology recommended anastrozole and outpatient follow-up visits. However, the patient and her family were hesitant with surgery, radiation, or chemotherapy given her age and comorbidities. She eventually declined the aggressive treatment of her breast cancer. She was bedridden upon discharge and passed away within 6 months after the initial presentation.

## Discussion

SPS is a rare neurological disorder with an estimated prevalence of one to two cases per million, affecting women two to three times more often than men. In classic SPS, rigidity and stiffness are usually symmetric and most prominent in axial and proximal limb muscles. They typically start in patients below the age of 50 and progress slowly over several years (2). However, in our patient, she developed asymmetrical stiff-leg syndrome (distal more than proximal) with coexisting myelopathy in her 80's, requiring the use of a wheelchair within 3 months from symptom onset. These unique clinical features have posed a diagnostic challenge to clinicians. The initial neurological localization is in the spinal cord, and differential diagnoses include myelopathy, atypical multiple sclerosis, motor neuron disease/primary lateral sclerosis, and focal dystonia. The EMG finding and the detection of anti-amphiphysin Ab are key to the diagnosis of paraneoplastic SPS in this case. Based on the diagnostic criteria by Dalakas (6), which include (1) muscular rigidity in the limbs and axial muscles; (2) continuous co-contraction of agonist and antagonist muscles, confirmed clinically and electrophysiologically; (3) episodic spasms; (4) absence of any other neurological diseases that could explain the symptoms; (5) and positive serology for GAD 65 (or amphiphysin) autoantibodies in the serum; the patient presented in this report met the criteria for diagnosis of SPS.

The pathogenesis of SPS is not fully understood. There is strong evidence that the impairment of GABAergic neurotransmission medicated by pathologic autoantibodies has caused lower GABA levels in the central nervous system (CNS) and has led to a loss of neural inhibition of skeletal muscles (6). The synaptic vesicle protein amphiphysin was discovered in 1992 by Lichte et al. (7). This protein is responsible for endocytosis of the vesicle membrane after the exocytosis of GABA from the axonal terminal (2). Anti-amphiphysin Abs are strongly associated with the paraneoplastic variant of SPS. Underlying cancer can be occult at the time and be diagnosed within years from the initial neurological symptoms. Rarely, underlying cancer can be detected after 5 years from the initial manifestation (8). In our patient, although a breast mass was noted about a decade before the onset of stiffness and spasms, the patient did not seek any medical attention. A decade-long progression of breast cancer followed by manifestations of SLS is atypical for paraneoplastic neurologic syndromes. The age of disease onset and the rapidly progressive course in our patient are distinctively different from those of classic SPS.

Experts used to believe that amphiphysin Ab-associated paraneoplastic SPS cannot be distinguished from classic SPS on clinical grounds since both groups have proximal muscle involvement (9). However, in a large case series (Yale SPS project) by Murinson et al. (10), 11 out of 621 patients had amphiphysin Ab-associated SPS, and all 11 patients had arm and neck involvement. Compared with classic SPS, amphiphysin Ab-associated SPS has been described to have a different pattern of stiffness, more likely to involve the arms and neck. In contrast to what is known in the literature, our patient had asymmetrical stiffness in the legs (predominantly in the distal legs) with dystonic posturing of the feet. McKeon et al. (11) have suggested that amphiphysin Ab-associated SPS should be considered in patients with stiffness and spasms confined to the extremities.

In amphiphysin Ab-associated SPS, additional clinical features including myelopathy, neuropathy, encephalopathy, and cerebellar ataxia have been described in case series (12), indicating that its clinical phenotype could be different from that of classic SPS. Our patient has myelopathic features including lower-extremity weakness and pyramidal tract sign. Considering the clinical course, coexisting paraneoplastic myelopathy is highly suspected. Although the brain and whole-spine MRI are unremarkable and infectious myelopathy is excluded through CSF studies, B12 and folate are normal; copper and vitamin E are not checked, which could be a limitation of our study. Her nerve conduction study shows evidence of sensorimotor axonal polyneuropathy, but there is a confounding factor of newly diagnosed diabetes (HbA1C 7.2).

Based on the pathogenesis, there are two main treatment approaches for SPS: first, the use of GABA-enhancing drugs and, second, immunomodulation. In a small randomized controlled trial with 16 patients who had SPS and anti-GAD Abs, intravenous immunoglobulins (IVIGs) were effective in reducing spasms and improving functional outcomes (13). A recent study suggested the safety and efficacy of therapeutic PLEX as an adjunct to immunosuppressive therapy in GAD Ab-associated SPS (14). However, in paraneoplastic SPS, randomized controlled trials are lacking due to the rarity of the disease. Anecdotal evidence suggests that amphiphysin-associated SPS may not respond to IVIG (15), and there are case reports proposing PLEX with steroids in this condition (16). In the Yale SPS project, Murinson et al. (10) suggested that amphiphysin-associated SPS is steroid responsive and that tumor excision with chemotherapy may produce marked clinical improvement. Our patient has received oral glucocorticoids for presumed “gout flare” before hospitalization without significant response; however, the exact dose and duration are unclear. The lack of response to steroids could be due to inadequate dosing or advanced disease.

Our patient responded to symptomatic management with diazepam (GABAa agonist), but not to immunomodulatory therapy with PLEX during the hospitalization. She declined aggressive cancer treatment including surgical resection as well as chemotherapy and passed away 2 months after discharge. This is consistent with the literature that the mainstay therapy in paraneoplastic neurologic syndromes remains the treatment of the underlying malignancies.

## Conclusion

SPS is a broad-spectrum disorder with heterogeneity in clinical phenotypes and associated Abs; it can be autoimmune or paraneoplastic. This case illustrates that the anti-amphiphysin SLS as a paraneoplastic neurologic syndrome associated with breast cancer can lead to devastating neurologic deterioration. This entity requires a high degree of suspicion for initiation of proper workup including neuroimaging, EMG/nerve conduction study (NCS), onconeural Ab testing, and cancer screening.

## Data Availability Statement

The original contributions presented in the study are included in the article/supplementary material, further inquiries can be directed to the corresponding author/s.

## Ethics Statement

Ethical review and approval was not required for the study on human participants in accordance with the local legislation and institutional requirements. The patients/participants provided their written informed consent to participate in this study. Written informed consent was obtained from the individual(s) for the publication of any potentially identifiable images or data included in this article.

## Author Contributions

BG produced the first draft of the paper and summarized the results. ES undertook the neurophysiology study and edited the paper. XF reviewed and edited the paper. JH helped with the pathology study and reviewed the paper. XL identified the case, revised, edited, and submitted the paper for publication. All authors made clinical and scientific contribution in writing the paper, read, and approved the final manuscript.

## Conflict of Interest

The authors declare that the research was conducted in the absence of any commercial or financial relationships that could be construed as a potential conflict of interest.

## Publisher's Note

All claims expressed in this article are solely those of the authors and do not necessarily represent those of their affiliated organizations, or those of the publisher, the editors and the reviewers. Any product that may be evaluated in this article, or claim that may be made by its manufacturer, is not guaranteed or endorsed by the publisher.
